# Considerations for Sex-Cognizant Research in Exercise Biology and Medicine

**DOI:** 10.3389/fspor.2022.903992

**Published:** 2022-06-03

**Authors:** Samia M. O'Bryan, Kathleen R. Connor, Devin J. Drummer, Kaleen M. Lavin, Marcas M. Bamman

**Affiliations:** ^1^Department of Cell, Developmental and Integrative Biology, The University of Alabama at Birmingham, Birmingham, AL, United States; ^2^UAB Center for Exercise Medicine, The University of Alabama at Birmingham, Birmingham, AL, United States; ^3^The Florida Institute for Human and Machine Cognition, Pensacola, FL, United States

**Keywords:** sex differences, exercise medicine, muscle, physiology, molecular biology

## Abstract

As the fields of kinesiology, exercise science, and human movement developed, the majority of the research focused on male physiology and extrapolated findings to females. In the medical sphere, basing practice on data developed in only males resulted in the removal of drugs from the market in the late 1990s due to severe side effects (some life-threatening) in females that were not observed in males. In response to substantial evidence demonstrating exercise-induced health benefits, exercise is often promoted as a key modality in disease prevention, management, and rehabilitation. However, much like the early days of drug development, a historical literature knowledge base of predominantly male studies may leave the exercise field vulnerable to overlooking potentially key biological differences in males and females that may be important to consider in prescribing exercise (e.g., how exercise responses may differ between sexes and whether there are optimal approaches to consider for females that differ from conventional approaches that are based on male physiology). Thus, this review will discuss anatomical, physiological, and skeletal muscle molecular differences that may contribute to sex differences in exercise responses, as well as clinical considerations based on this knowledge in athletic and general populations over the continuum of age. Finally, this review summarizes the current gaps in knowledge, highlights the areas ripe for future research, and considerations for sex-cognizant research in exercise fields.

## Introduction

### Historical and Current Sex Difference Gaps in Exercise Research

Throughout its history, exercise research has been both driven and dominated by a predominantly male population. Credited with the origin of the modern exercise science field, the Harvard Fatigue Laboratory (early-mid 20th century) academics conducted exercise research on primarily male lab members and athletes (Chapman, 1990; Scheffler, 2015). Additionally, in response to data of poor physical fitness in World War I and II draftees, physical education became a nationwide focus, as President Kennedy enacted initiatives to rescue fitness deficits in American boys and girls. Disciplines distinct from athletics were established in the form of kinesiology, exercise science and human movement (Berryman, 2010).

It was not until the enactment of Title IX in 1972, which banned sex discrimination in any federally funded education program, that females began to enter sports in larger numbers (Costello et al., 2014). Unfortunately, in keeping with medical and drug research trials, females were often excluded from exercise research studies due to the perceived complexities of the menstrual cycle or the misguided notion that exercise could potentially harm unborn fetuses (Bruinvels et al., 2017). Consequently, findings from male-dominated studies were extrapolated to females. In addition, the US National Institutes of Health (NIH) guidelines at the time urged for female inclusion without requiring it, which allowed researchers to continue to conduct major clinical trials without female inclusion (Schiebinger, 2003). Further, when studies did include females, they were not analyzed in a manner to determine sex differences (Schiebinger, 2003; Mazure and Jones, 2015). Thus, the NIH Revitalization Act of 1993 was introduced, mandating female participation in Phase III clinical trials (National Institutes of Health Revitalization Act of 1993, 1993). Unfortunately, not all trials have strictly adhered to these guidelines since inception. The consequences of ignoring sex differences in favor of “clean” research became abundantly clear in a letter from the U.S. General Accounting Office in 2001 where an audit of pharmaceuticals removed from the drug market since 1997 discovered 80% of the removals (i.e., antihistamine, cardiovascular, and gastrointestinal drugs) were due to severe adverse effects in females (United States General Accounting Office, 2001). At the same time, a short review concluded that being female was a strong risk factor to developing adverse drug reactions (Rademaker, 2001). For example, non-steroidal anti-inflammatory, cardiovascular, general anti-infectives, nervous system and musculoskeletal drugs were among those for which up to 70% of reported adverse drug reactions occurred in females (Rademaker, 2001).

While it is generally understood that exercise confers a host of important health benefits and is well tolerated by most, differential responses among individuals and groups do occur, and understanding the sources of these differences is important in order to optimize exercise prescription for the individual. In this context, biological sex is an important factor to consider. Unfortunately, a 2014 study found that of 1,382 original human exercise medicine and sport research articles (2011–2013) including over 6 million participants, only 39% of participants were female (Costello et al., 2014). In molecular exercise physiology research, a potentially greater disparity prevails: among >60 studies recently compiled to build a database of skeletal muscle gene expression in response to acute and chronic exercise, only ~25% of participants of all studies were female, and many studies failed to report sex (31% of resistance trained participants were undefined) (Pillon et al., 2020). Whether this disparity has existed and persisted due to societal gender norms, lack of resources or interest among females, or other influences is less important than what is left in the aftermath: that much of our understanding of exercise physiology is primarily limited to knowledge of male responses and adaptations. A separate but related problem is that research in exercise metabolism often tests females when hormones such as estrogen are at their lowest, i.e., when females are most “male-like” (Bruinvels et al., 2017; Vilhena, 2017; Sims and Heather, 2018; Wee et al., 2018; Taipale et al., 2020). Ignoring the fundamental and dynamic roles sex hormones may play in response to exercise throughout the menstrual cycle serves only to perpetuate the sex disparity in exercise research (Bruinvels et al., 2017; Sims and Heather, 2018).

### Considerations for Sex-Cognizant Research in Exercise Biology and Medicine

While we acknowledge there is a spectrum of societal gender identity, in this review we focus only on biological sex (i.e., male and female). However, some may consider even biological sex is not truly binary (Johnson et al., 2009). For example, females more often show a wider variability of characteristics within the female sex, likely due to differences in hormone concentrations among females (Institute of Medicine Committee on Understanding the Biology of Sex Gender Differences, 2001; Johnson et al., 2009). Furthermore, some biological conditions may predispose individuals to exhibiting non-binary characteristics, (i.e., chimeras, XXY, XYY, XO) that may not be immediately identifiable and contribute to intersex variability in research (Johnson et al., 2009). Ultimately, due to the variability within sexes, overlap between the sexes is possible, particularly in regards to sex hormones (e.g., estrogen, testosterone) resulting in “male-like” females and vice versa, which may further contribute to masking true sex differences.

A current barrier to understanding sex-specific exercise responses is that many studies rarely have the statistical power due to an overall low participant number or limited female recruitment to truly interrogate sex differences. In addition, sex-specific reporting policies instated by journals may be a faulty approach, as this can result in underpowered analyses and over-interpretation of results which muddles the literature on sex differences (Wizeman, 2012). Instead, studies underpowered for sex difference analyses should be recommended to include participant data specific to sex and exercise response parameters as supplementary material in order to facilitate future meta-analyses and prevent erroneous, over-inflated interpretations of sex specific exercise responses. Furthermore, the typical statistical approach to analyzing groups of males and females within a study has been to “control” or “correct” for sex to remove sex-associated variance and apply findings universally to both sexes. This is a statistically inappropriate way to approach sex differences (Beltz et al., 2019), especially since sex differences are considered non-random variance (Miller and Chapman, 2001). This further supports pushing for equal recruitment of both sexes in exercise research so that at the very least, stratified analyses can be done to identify potential sex-based differences.

A limitation in sex differences analysis is that females are typically compared to males and male-derived data. Historically, male data were derived from comparisons to other males, establishing males as a “reference” or “control” population to which female populations were later compared. However, female-to-female comparisons are less understood due to the lack of such studies and difficulty in understanding differences when traits are measured on a scale developed for only one sex (Beltz et al., 2019). Instead, stratified analysis of sex by groups would allow for both sex-difference analysis and the establishment of female-specific scales. This would enable researchers to elucidate sex differences at the molecular level in response to exercise, contributing to a deeper understanding in physiological changes that may impact exercise and disease responses, drug targeting, aging, etc. In addition, bolstering the current literature with stratified analyses, and some female-specific studies where appropriate, would be informative to understanding the most effective approaches in exercise within female cohorts at the functional and molecular levels.

**Figure 1:**
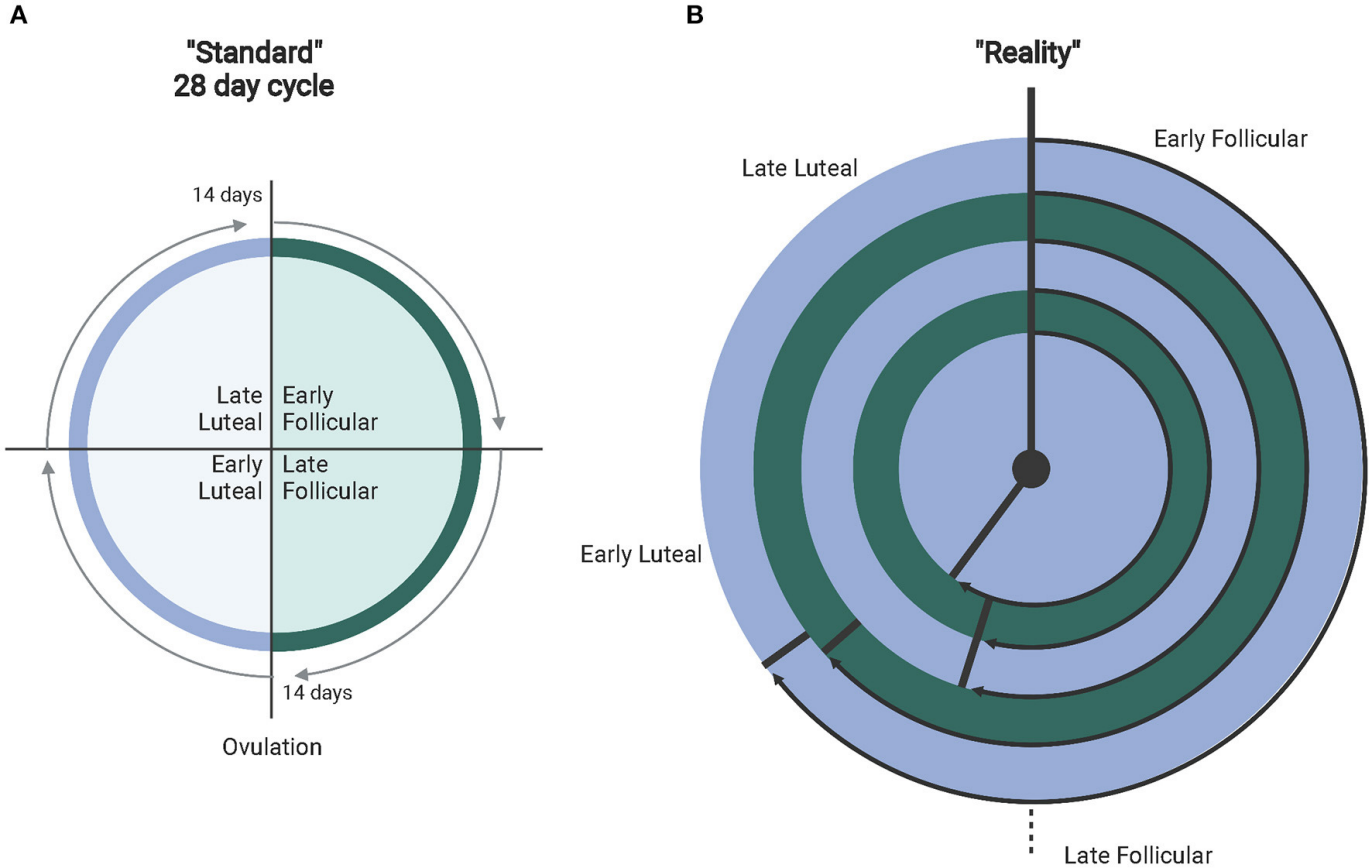
**(A)** Depicts the standardly accepted 28-day menstrual cycle. **(B)** The different ranges of real-world normal cycles (experiencing proper hormone fluctuations). From outer to inner ring: 36–50 days (avg. 40 depicted) with ovulation at 26.8 days (67% follicular). 31–35 days (avg. 32 days)–ovulation at 19.5 days (61% follicular). 25–30 days (avg. 28 days follicular) with ovulation at 15.2 days (54% follicular). 21–24 days (avg. 23 days) with ovulation at 12.4 days (54% follicular). Finally, 15–20 days (avg. 18 days) with ovulation at 10.4 days (57% follicular). Created with BioRender.com.

Other important considerations in interpreting or conducting exercise research with females include whether they are eumenorrheic, using a form of hormone-based contraception, and when during the menstrual cycle each dataset is collected. Of note, it is important to understand that the presence of a “regularly presenting” cycle does not confirm that ovulation occurs (anovulation) (Abraham, 1978; de Janse, 2019). Additionally, the length of the menstrual cycle can vary, along with varying lengths of the menstrual cycle phases (Figure 1) (Fehring et al., 2006; Mihm et al., 2010; Bull et al., 2019). This suggests that simply recording first and last day of a menstrual period and the amount of time between each is insufficient data when considering the effects of the menstrual cycle on exercise responses. Identifying specific factors (i.e., hormonal concentrations, temperature) will help research stream-line analyses in females during hormonal fluctuations and as a result produce more cohesive bodies of research that can be utilized for real-world application of exercise regimens. Collecting these types of data for publication is essential for future meta-analyses where limitations can be minimized and power maximized. In addition, addressing the complexity of the menstrual cycle in research may no longer be as complicated as it has been suggested in the past, particularly when considering pairing hormonal testing with wearable technology (i.e., Oura Ring) (Maijala et al., 2019). and FDA-approved menstrual cycle tracking apps (i.e., Natural Cycles, Clue) (Berglund Scherwitzl, 2015; Moglia et al., 2016; Berglund Scherwitzl et al., 2017). that utilize temperature and menstrual period dates to track and predict menstrual cycle phases.

Overall, while a number of biological responses to exercise may overlap between the sexes, emerging research indicates many do not; thus it is critical to consider the effects of male and female physiology separately, particularly with regard to underlying molecular phenomena that may drive key processes related to exercise, health, disease, and drug responses. The assumption that findings from males apply to female populations may cause us to overlook important sex differences and fail to optimize treatment strategies to promote health. Ultimately, understanding the basis for sex differences in exercise will lead to the best possible approach for targeted exercise prescriptions. Within this review, we highlight data primarily from the last 15 years to highlight recent advances that may need more research. Earlier studies were included if they were pertinent to the studies being discussed. Studies chosen for inclusion were found through PubMed and Google Scholar based primarily on keyword searches: sexual dimorphism/sex differences, muscle, human, and exercise.

### The Physiological Impact of Hormones on Muscle and Performance

#### Testosterone

A large contributor to physiological differences between males and females is endogenous density and production the sex steroid receptors and their hormones estrogen, progesterone, and testosterone. Prior to puberty, males do not exhibit a performance advantage over females. After the onset of puberty, males exhibit a marked divergence in performance resulting in improved performance in aerobic and power events (Handelsman, 2017; Handelsman et al., 2018). This has been attributed to a rise in testosterone, an androgen that remains fairly consistent day to day during the reproductive lifespan (Handelsman et al., 2018). As a result, testosterone has been strongly associated with the greater muscle mass and strength observed in males post-puberty when compared to females (Handelsman, 2017). Females with hyper-androgen disorders, such as polycystic ovary syndrome (PCOS), have also demonstrated better performance and higher muscle mass and power than non-PCOS females (Douchi et al., 2001; Rickenlund et al., 2003; Eklund et al., 2017).

**Figure 2:**
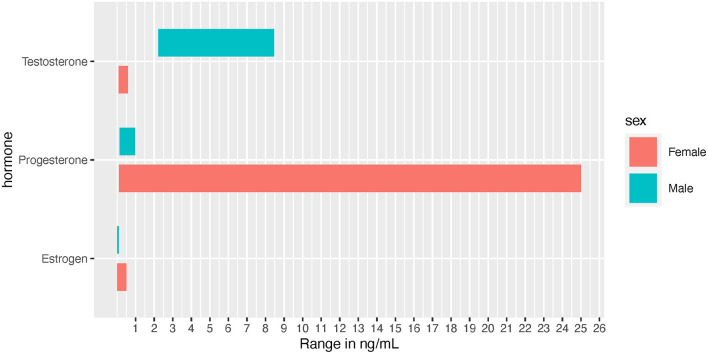
Normal hormone concentration ranges depicted in ng/mL for visual comparison between the sexes. Female ranges depict the low to high values experienced throughout the menstrual cycle.

Due to the effects on male performance and muscle enhancement, androgens and the androgen receptor (AR) have been extensively studied. Androgens exist in two bioactive forms, testosterone and dihydrotestosterone (DHT), which are released from the testes, ovaries, and adrenal glands (Miller and Auchus, 2011). The primary focus here is testosterone, which typically circulates at 7.7-29.4 and <2 nmol/L in adult males and females, respectively (Figure 2) (Handelsman et al., 2018). The effects of circulating testosterone are primarily mediated by ARs, which are nuclear receptors located in the cell cytosol that translocate to the nucleus upon ligand binding. In skeletal muscle (SkM) satellite cells and myofibers testosterone-mediated AR activation induces muscle satellite cell proliferation, myofiber hypertrophy, and myonuclear number (Sinha-Hikim et al., 2003; Herbst and Bhasin, 2004; Kadi, 2008). In addition, a recent study with recreationally active females, testosterone was administered to moderate levels (~4.65 nmol/L) for 10 weeks, resulting in type II myofiber hypertrophy, an expansion in the microvascular network and increases in satellite cell number, although myonuclear number was unchanged (Horwath et al., 2020). In males, testosterone administration (exogenous; at supraphysiological and physiological doses) is widely understood to induce muscle hypertrophy [reviewed in Bhasin et al. (2003)] while endogenous free testosterone levels are associated with lean mass levels (Mouser et al., 2016).

Testosterone is clearly a major sex-related variable that affects the molecular exercise response driven by several fold differences in biological concentrations between males and females. While exercise training does not increase basal levels of testosterone, some recent evidence suggests that females have a similar acute biphasic response (albeit different sex-dependent concentrations, i.e., 3.18 ng/dL (males) vs.146 ng/dL (females) 24 h post-exercise) in testosterone as males after intensive, prolonged endurance training (ET) (Anderson et al., 2016; Hackney and Willett, 2020). However, there are no direct linkages of acute changes in testosterone to long-term training adaptations and the physiological role of this rise in testosterone in response to exercise in females remains even less understood. It is more likely that an individual's basal level is more influential in training adaptions.

#### Estrogen

Effects of estrogens on SkM mass are not clear (Enns and Tiidus, 2010; Hansen and Kjaer, 2014), although some evidence points toward muscle mass retention in post-menopausal females receiving hormone replacement therapy (Tiidus, 2003). In pre-menopausal females, estrogens and progesterone are produced by the ovaries in a cyclical fashion over a 15–50 day period, with an average of a 29.3 day cycle (Bull et al., 2019). Over the course of the menstrual cycle the most prevalent estrogen (i.e., 17β- estradiol), can fluctuate from 5 pg/mL to 500 pg/mL, (Chidi-Ogbolu and Baar, 2019) while males exhibit circulating levels of 3.6–91 pg/mL (Figure 2) (Cooke et al., 2017). Estrogens typically prime tissues to respond to progesterone, whereas progesterone usually decreases tissue response to estrogens (Sims and Heather, 2018). In addition, the human genome contains over 70,000 estrogen responsive elements interconnected in a complex web of gene regulation and expression, likely driven by tissue type and tissue responsiveness as an estrogen target (Bourdeau et al., 2004). Within muscle tissue, estrogens influence blood flow and blood pressure regulation. In part because estrogens help mediate greater nitric oxide synthase (NOS) production, females often experience higher vasodilation in response to exercise (Parker et al., 2007; Gavin et al., 2009; Kellawan et al., 2015; Joyner et al., 2016). However, while NOS may contribute temporarily to increased vasodilation during the luteal phase, the majority of sex differences seen in SkM vasodilation may be independent of NOS (discussed in Kellawan et al., 2015).

Like ARs, estrogen receptors Erα, which is the predominant estrogen receptor (ER) (Ekenros et al., 2017), and ERβ partake in canonical nuclear signaling like ARs to facilitate biological responses. Their actions following ligand binding appear to help protect against exercise-induced muscle damage (Tiidus, 2000; Dieli-Conwright et al., 2009; Pal et al., 2018) and muscle cell apoptosis (Vasconsuelo et al., 2008; Hevener et al., 2020), and improve mitochondrial function (Hevener et al., 2020). Estrogen receptor-related receptor isoforms ERRα and ERRγ are highly expressed in SkM and play key roles in increasing oxidative capacity and fatty acid utilization (Huss et al., 2015). ERRα expression has been shown to increase in SkM in response to exercise (male data) (Cartoni et al., 2005), leading to estrogen-mediated signaling that results in increased ability to store glycogen in muscle while increasing free fatty acid availability and use through upregulation of oxidative pathways (Nicklas et al., 1989; Hackney, 1990; Sims and Heather, 2018). This shift in substrate utilization results in decreased reliance on carbohydrates (Oosthuyse and Bosch, 2010, 2012). In addition, estrogens may play a role in muscle size, acting as a protective mechanism against muscle atrophy, a correlation that can be seen when estrogen declines during aging and some muscle myopathies (Baron et al., 2011; Carson and Manolagas, 2015). Unfortunately, the vast majority of mechanistic studies related to muscle response and estrogen have been conducted in animal models. While informative, particularly in highly conserved pathways, estrogen can have variable effects specific to species due to differences in reproductive cycles (Hansen and Kjaer, 2014), resulting in lower translational power and making it difficult to predict whether human SkM responds in the same manner. Additionally, much of the research in estrogen and exercise response has been reported as a result of sex difference analyses in mixed sex studies, confounding the data with male vs. female results. Thus, estrogen effects on muscle hypertrophy in various conditions in human, female-only cohorts needs to be further explored.

#### Progesterone

Progesterone is typically discussed in regards to females, as female adults can have concentrations ranging from 0.89 to 25 ng/mL throughout the menstrual cycle, while adult males are approximately at 0.20 ng/mL (Figure 2) (Rifai, 2017). Progesterone often acts in opposing mechanisms to estrogens: for example, it can inhibit estradiol's effects on carbohydrate metabolism (D'Eon et al., 2002). Based on earlier research, it was suggested that progesterone continues to act in opposition to estrogen by promoting catabolism and reducing muscle protein synthesis (Kalkhoff, 1982; Lamont et al., 1987). However, in contrast to this effect, a study examining sedentary pre- and post-menopausal females found that progesterone resulted in an increased rate of protein synthesis independent of testosterone and estradiol activated pathways (Smith et al., 2014). However, the effect of progesterone on human SkM mass has not been evaluated directly. It is noteworthy to mention that a truncated progesterone receptor has been discovered (in cardiac muscle) to localize to the mitochondrial membrane, suggesting that progesterone may act directly on the mitochondria. If this finding is recapitulated in human SkM, there may be further exercise-associated sex differences in SkM mitochondrial metabolism perpetuated by this non-nuclear pathway (Shah et al., 2013).

While evidence of menstrual phase effects on muscle-specific exercise response is contradictory, some points toward shifts in full body responses to exercise. For example, when comparing follicular vs. luteal phases over the course of five menstrual cycles or 4 months of RT; strength, power, muscle hypertrophy and cellular responses are greater during the mid-late follicular phase (peak estrogen) vs. the luteal phase (Sung et al., 2014; Wikström-Frisén et al., 2015). Conversely, progesterone during the luteal phase may contribute to decreased athletic performance, partially due to its thermoregulatory effects. During this phase, heart rate, (Sedlak et al., 2012) basal body temperature and ventilation increase (Charkoudian et al., 1999), which can result in the perception of increased exertion (*via* RPE, thermal comfort scale, sweat perception and heart rate), thus contributing to decreased exercise performance seen in hot and humid conditions (Janse De Jonge et al., 2012). Specifically, in humans, endogenous estrogen has been suggested to have anabolic effect, possibly contributing to hypertrophy (although no direct links have been established), while progesterone is suggested to have a catabolic effect (Lamont et al., 1987). However, this evidence remains inconclusive due to evidence of progesterone (exogenous) stimulating protein synthesis in post-menopausal females. Thus, a more thorough understanding of progesterone's mechanisms of action within muscle is needed (Rosa-Caldwell and Greene, 2019).

#### Thyroid Hormone

The thyroid gland is among the tissues with the largest number of differentially expressed and targeted genes by sex (Lopes-Ramos et al., 2020). SkM is a major target for thyroid hormone (TH). For example, SkM contractility, as a measure of ATP turnover rate in humans has been shown to differ based on thyroid hormone status (Wiles et al., 1979), as individuals with hyperthyroidism exhibit higher ATP turnover rates and hypothyroid individuals have lower ATP turnover rates (Wiles et al., 1979). How this may contribute to sex differences is seen in overall SkM changes due to thyroid status. Outside of animal studies, which show that TH can alter fiber-type composition (Haizlip et al., 2015), evidence in humans has shown that hypothyroidism and hyperthyroidism can impact fiber type to different extents in males and females. For instance, females with hypothyroidism not only tend to have more type II fibers but also experience type II fiber atrophy when compared to hypothyroid males (Haizlip et al., 2015). In euthyroid individuals, strenuous exercise may result in transient decreases in TH post-exercise, although this does not always occur and whether sex differences in exercise TH responses occur under non-pathological conditions (Hagobian et al., 2009), is not clear (sparse literature in this area).

## Considering Sex Differences in Tissues for Exercise Research

### Body Composition

Beginning in puberty, body fat distribution differs between males and females, suggesting that hormones are the primary drivers of this patterning (Wells, 2007). In brief, females tend to have higher fat mass over the course of a lifespan, requiring a normal physiological function reserve of essential fat of 12% when compared to 3% in males (Flynn et al., 2018). Specifically, females tend to store fat in the lower body, and excess fat tends to be deposited subcutaneously (White and Tchoukalova, 2013). In contrast, males tend to store fat in the trunk of the body, and excess fat is typically stored around the organs (viscerally) (Karastergiou et al., 2012). These differences in body fat distribution may be in part driven by estrogen levels, as visceral adipose is low in ER and subcutaneous adipose has higher levels of ER (Brown and Clegg, 2009). Additionally, androgens (i.e., testosterone) play a role in fat mass distribution and accumulation. For example, androgens have been shown to inhibit human (male and female) pluripotent mesenchymal stem cells and preadipocytes from differentiating into an adipocyte lineage while simultaneously promoting myogenesis (Bhasin et al., 2003; Gupta et al., 2008; Blouin et al., 2010). This evidence is further supported by the reversal of fat accumulation in hypogonadal males with the addition of androgen therapy (Bhasin et al., 2003). In contrast, studies in both murine and human cell cultures demonstrate that estrogen stimulates adipogenesis (Roncari and Van, 1978; Anderson et al., 2001), while simultaneously regulating adipose mass and accrual (Van Pelt et al., 2015).

SkM mass differs between the sexes as well. Typically, males have more SkM in absolute terms and relative to body mass, and males tend to carry a greater percentage of SkM in the upper body, and a lower percentage of SkM in the lower extremities compared to females (Janssen et al., 2000). On average, among females vs. males, upper arm and thigh muscle cross-sectional areas are 50–60% and 65–70%, respectively, of their male counterparts (Handelsman et al., 2018). Consequently, sex differences in strength are greater for the upper (~40–50%) vs. lower (~30–40%) body between males and females (Miller et al., 1993; Handelsman et al., 2018).

### Bone and Tendon

The relationship between SkM and bone is not purely mechanical, but also includes complex molecular communication resulting in different phenotypic and functional outcomes. Sex differences in the bone-muscle relationship begin to emerge during adolescence when sex hormones begin to influence growth (Zofkov, 2008). Notably, males develop a thicker periosteum while females develop a thicker endosteum that drives increased cortical mass (Kontulainen et al., 2006; Zofkov, 2008). Exercise is important for bone remodeling as exercise-related loading induces stress within bone and thus stimulates osteoblast activity (Santos et al., 2017). However, with a limited research base, it is not clear whether there are significant bone health differences between adult pre-menopausal females and age-matched males (Santos et al., 2017).

A recent study highlighted that the muscle-tendon complex adaptation to resistance training (RT) differs in young females and males (McMahon et al., 2018). Specifically, patellar tendon adaptation to RT, as characterized by RT-induced stiffness, Young's Modulus, and the tendon force-elongation curve, was greater in young females at a lower percentage (10–20%) of their maximal voluntary contraction (MVC), whereas, in males greater adaptation was seen at higher percentage (90–100%) of their MVC (McMahon et al., 2018). This suggests that a modified RT training approach to strengthen and optimally adapt the female patella tendon to RT might be more successful when done at a lower percentage of their MVC, although more research is needed.

### Muscle Morphology and Function

The general consensus is that females tend to have a greater number and percentage of type I myofibers over Type II in mixed fiber muscle (i.e., vastus lateralis) when compared to males (Simoneau and Bouchard, 1989; Miller et al., 1993; Steffensen et al., 2002; Walker et al., 2012; Lundsgaard and Kiens, 2014; Roberts et al., 2018). Some evidence suggests that sex differences in fiber type distribution are not significantly different when looking at moderately vs. long-term (2+ years) highly trained (endurance) individuals (Steffensen et al., 2002). Other evidence suggests that fiber type distribution shifts in response to resistance exercise (reduction of Type I fibers and an increase of Type II fibers), but ultimately does not result in significant sex difference (Moro et al., 2020). Furthermore, a study looking at short term (7 wk) endurance training found no significant changes to fiber type distribution in either sex (Carter et al., 2001b), while others show a switch within Type II fibers from glycolytic (IIx) to oxidative (IIa) in response to endurance (reviewed in Yan et al.) and resistance training (Kosek et al., 2006; Martel et al., 2006). Unfortunately, there remains a lack of sex difference comparisons of fiber type distributions in long term exercise approaches. Regardless, SkM fibers are quite plastic and responds to type of training (endurance vs. resistance) through neural and signaling pathways, resulting in fiber-level changes to distribution and size (Talbot and Maves, 2016).

**Figure 3:**
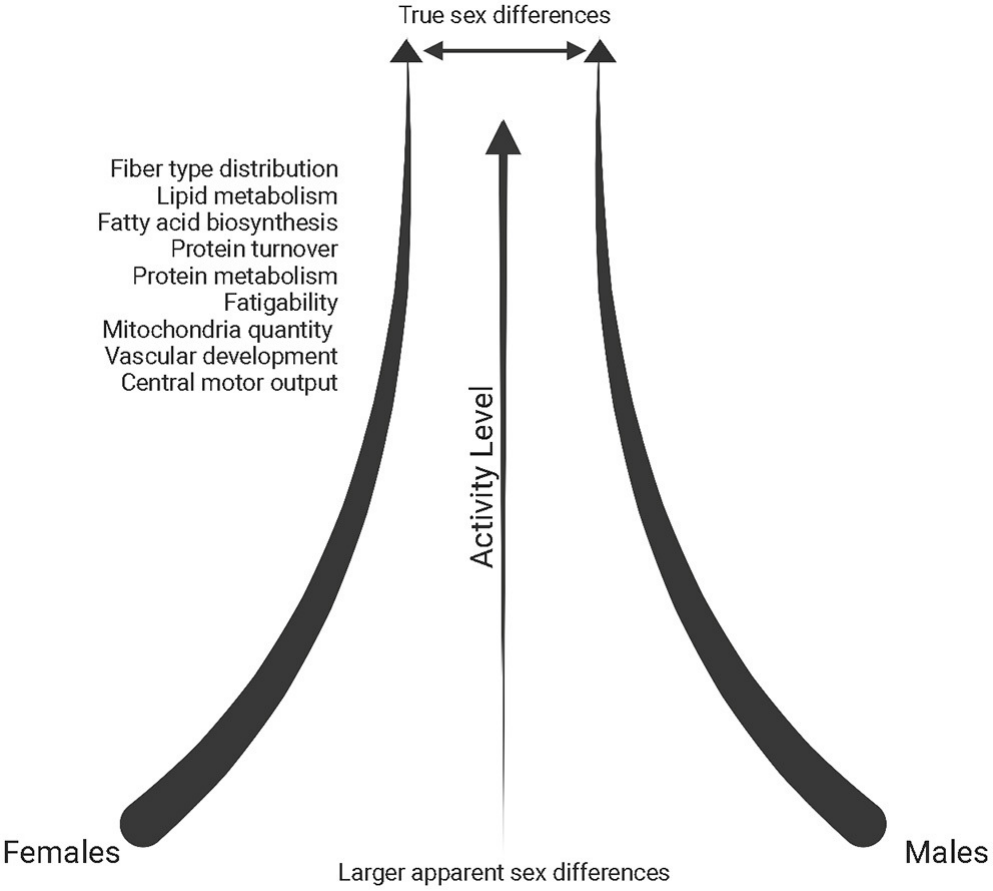
Schematic of the reduction in sex differences reported in the literature (*throughout text*) with increasing physical activity levels. Created with BioRender.com.

The muscle environment itself can translate into some mechanical differences in muscle function. For example, Type I muscle fibers have a slower rate of relaxation after contraction due to differences in calcium kinetics between fiber type (Wüst et al., 2008; Lamboley et al., 2014). As a result, there are demonstrated sex differences in muscle fatigability if contractions are performed at the same relative intensity (reviewed in Hunter, 2016). This could in part be due to greater muscle perfusion and the ability of females to remove metabolites, such as inorganic phosphate, out of working muscle more efficiently (Kent-Braun et al., 2002; Kellawan et al., 2015; Hunter, 2016). However, sex differences in fatigue are not consistently found (Kent-Braun et al., 2002; Russ and Kent-Braun, 2003; Russ et al., 2005). This seems to be particularly the case in physically active and athletic individuals, where sex differences are often diminished, perhaps due to fiber-type composition becoming closer to homogeneous across sexes in trained conditions (Cairns et al., 2017), possibly resulting in a decrease of fatigability in males (Figure 3).

Males tend to have larger type I and type II myofibers vs. females (Miller et al., 1993; Staron et al., 2000). However, number of fibers per motor unit (MU) and number of MUs do not differ between the sexes in adults (19–31 years) (Miller et al., 1993). The sex difference in force generation capacity is thus driven largely by differences in myofiber size, not number. At a single fiber level, sex differences in specific force (maximal isometric force normalized to CSA) have been shown only in type IIx fibers, although these data are very limited (Jeon et al., 2019). Interestingly, initial MU firing rates have been shown to be greater during the ovulation, mid-luteal and late luteal phases of the menstrual cycle when compared to the earlier phases (early, late follicular) (Tenan et al., 2013). More research is needed to understand how differences in MU firing may translated to differences in functional performance between the sexes and across the menstrual cycle.

### Molecular Underpinnings of Skeletal Muscle Response to Exercise

In addition to muscle fibers, SkM includes other cell populations that contribute to muscle growth and function, including: immune cells, endothelial cells, and muscle stems cells (satellite cells) (Bentzinger et al., 2013). It is important to note that the SkM milieu is different in different muscles (Micheli et al., 2020) and the majority of research in humans focuses on the vastus lateralis, with few exceptions. While there are significant gaps in knowledge, this section will highlight what is known and point to potential sources of sex differences.

#### SkM Cellular Milieu

Satellite cell activation, number, and differentiation can be impacted by sex. While both testosterone and estrogen display anabolic roles through the activation of satellite cells, testosterone tends to promote later stages of myogenesis through differentiation and growth (Sinha-Hikim et al., 2003, 2006; Velders and Diel, 2013), while estrogen plays a greater role earlier in myogenesis through stem cell activation and proliferation in conjunction with macrophages and ultimately protects against catabolic processes (Collins et al., 2019; Luk et al., 2019). However, these mechanisms are not fully understood and contradictory results have been reported, such as an absence of satellite cell proliferation perhaps due to greater cortisol impact in females in response to RT (Luk et al., 2019). In this case, cortisol (catabolic) rises similarly in males and females after RT, however, it antagonizes estrogen receptors potentially having a catabolic effect in females perhaps due to less testosterone which counteracts cortisol (Luk et al., 2019). On the other hand, there is evidence that males and females respond similarly in increasing satellite cell numbers to chronic resistance training (8 weeks) (Sawan et al., 2021). A recent meta-analysis also reported on the lack of sex differences in increases in fiber cross sectional area and myonuclei number, although this could be a result of pooling data from individuals aged 17–60 and 60+ years old (Conceição et al., 2018). Regardless, most mechanistic studies on both testosterone and particularly estrogen are confined to animal studies (Colla et al., 2015) and it is necessary to further explore this in humans *in vivo* and *in vitro*.

Muscle progenitor cells (MPCs) are committed myoblasts central to repairing damaged SkM by promoting the formation of myotubes (Chargé and Rudnicki, 2004). Inhibiting or decreased MPC expansion capability may translate into reduced muscle regeneration and hypertrophy (Riddle et al., 2018). Interestingly, in a cohort of young and old individuals that reported exercising more than 2 days per week (resistance and endurance), the expansion capacity in MPCs from young females was lower than those from young males (Riddle et al., 2018). However, where male MPC expansion capacity declined with age, female MPC expansion capacity was preserved (Riddle et al., 2018). These differences may be, in part, due to decreases in cell cycling and increases in cell death among female MPCs (*in vitro*) when compared to male MPCs (Riddle et al., 2018).

#### Epigenetic, Transcript and Protein Differences

Studies most often focus on the acute effects of exercise to determine the epigenetic transient changes that influence muscle adaptation while others look at long-term changes in response to exercise. A few studies have shown differential changes between the sexes with females exhibiting a more dynamic and robust response in methylation changes due to exercise; however, a deeper dive into specific sex differences in methylation and subsequent downstream effects is sorely needed (Lindholm et al., 2014b; Brown, 2015).

Many molecular studies in humans have focused on *in vitro* SkM cell analyses. For example, a study by Davegårdh et al. (2019) reported methylation and gene expression patterns persisting from myoblasts to myotubes related to and independent of the X-chromosome. Specifically, Lysine Demethylase 6A (KDM6A), responsible for removing repressive histone marks in muscle-specific genes was found to have decreased methylation and higher expression in females (training status unknown) (Davegårdh et al., 2019). This may be a factor in higher expression levels of oxidative pathway genes that support differentiation and regeneration. On the other hand, higher expression of TGF-beta in muscle cells of males suggests the promotion of myoblast proliferation over differentiation (Davegårdh et al., 2019). In addition, DNA damage checkpoint control and apoptosis gene expression are increased while cyclins and cell cycle control genes are decreased in female MPCs compared to male MPCs, suggesting female cells have reduced expansion capacity, at least *in vitro* (Riddle et al., 2018).

In a study by Lindholm et al. (2014a) baseline SkM gene expression analysis of sedentary young adults found that females exhibited enriched gene expression associated with metabolism of oxo- and carboxylic acids, ketones, and fatty acids as well as cellular respiration and oxidation reduction, while males had higher expression of genes enriched in pathways related to protein catabolism (Lindholm et al., 2014b). In addition, endothelial markers [vascular endothelial growth factor receptor 2 (KDR), endothelial tyrosine kinase (TEK), vascular endothelial growth factor receptor 1 (FLT1), Fms-Like tyrosine kinase 4 (FLT4)] and angiogenic factor transcripts [vascular endothelial growth factor A (VEGFA) isoforms, angiopoietin 1 (ANGPT1), fibroblast growth factor receptor 1 (FGFR1)] were enriched in females, congruent with higher capillary density associated with type I fibers (Lindholm et al., 2014b). Similarly, significant sex differences have been observed in transcriptional analysis of muscle from sedentary controls. Specifically, sedentary males had differentially regulated genes at baseline that suggested enhanced protein ubiquitination, catabolic, and ribosome biogenesis pathways, whereas females demonstrated gene expression for the enhancement of pathways associated with extracellular structure, wound healing and lipid metabolism (Chapman et al., 2020). It is important to remember that transcriptional data (mRNA abundance) can be inconsistent with protein expression and correlating these types of data to various molecular levels (i.e. miRNA, methylome) may be necessary to understand the true extent of the molecular response to exercise.

With respect to the exercise response, one of the earliest large-scale exercise-based SkM gene expression studies in humans showed a handful of differentially expressed genes between sexes at baseline and in response to strength training, specifically: ankyrin 2 (ANK2), mitogen-activated protein kinase kinase 12 (MAP3K12), protein phosphatase 1 (PP1) and Ras homolog family member H (RhoH), although the effects of these biomarkers were not specifically explored (Roth et al., 2002). In a study that utilized *biceps brachii* biopsies, the female participants demonstrated activation of TGF-beta and Notch signaling, as well as upregulation of genes related to SMAD binding suggesting that the hypertrophic effect may be attenuated in comparison to the male participants which exhibited significant upregulation of mTOR signaling (based on same relative intensity) (Liu et al., 2010). When compared to the previous *in vitro* study above (Davegårdh et al., 2019), it seems experimental differences may result in differential expression of genes related to TGF-beta signaling, perhaps due to the removal of circulating hormones *in vitro* vs. *in vivo*, and therefore there needs to be more exploration to fully understand the clinical significance of such biomarkers *in vivo* or a more concentrated effort to recapitulate the hormonal milieu *in vitro*.

In an analysis of differentially expressed transcripts in endurance-trained individuals (minimum 15 years of training), females exhibited upregulation of protein-turnover associated genes and males had upregulated expression of oxidative metabolic pathways and peptide metabolism, yet overall, sex differences within the endurance-trained group were significantly diminished when compared to sedentary controls (Figure 3) (Chapman et al., 2020). However, an analysis between sedentary and endurance-trained males uncovered enhanced expression of genes associated with fatty acid biosynthesis, oxidative metabolism and mitochondrial structure while a comparison between sedentary and endurance-trained females uncovered enhanced expression of genes involved in oxidative metabolism, vascular development and protein metabolism pathways (Chapman et al., 2020).

#### Metabolic

As an integral component of metabolic function, mitochondrial function has been extensively explored in animal models, consistently showing females exhibit greater oxidative capacity, lower ROS production, increased fatty acid utilization and mitochondrial respiration regulation *via* 17-β estradiol (estrogen) (Torres et al., 2017; Ventura-Clapier et al., 2017). In some cases, these data have been upheld in humans, demonstrating that brain and adipose mitochondria have higher functional capacity in females (Harish et al., 2013; Nookaew et al., 2013). However, the role of sex and its interaction with a genetic background on mitochondrial function in human SkM has been far less explored and whether similar sex differences exist is yet to be fully determined.

As mentioned previously, females typically have a greater distribution of type I fibers than males, and animal studies suggest that estrogen represses mitochondrial uncoupling protein (Ucp3), thus preventing energy dissipation and supports more efficient energy production (Ikeda et al., 2019). In addition, many studies have determined that females (human and animal) typically have greater mitochondrial content per gram of tissue than males, suggesting intrinsic differences in mitochondrial function between the sexes (Rosa-Caldwell and Greene, 2019).

A few recent studies report that oxidative phosphorylation capacity (per unit muscle mass) is similar in males and females. However, decreased mitochondrial adenosine diphosphate (ADP) sensitivity has been observed in females when compared to males, which is consistent with animal studies and has been correlated with percent body fat and greater physical fitness in females (Ferreira, 2018; Miotto et al., 2018; Montero et al., 2018). On the other hand, lower mitochondrial O_2_ consumption observed in females (Miotto et al., 2018) would require more ADP accumulation to achieve the same VO_2_ as males at matched workloads (Ferreira, 2018). However, higher inorganic phosphate levels may then interfere with muscle fiber function, which may contribute to challenges in endurance performance (Ferreira, 2018; Miotto et al., 2018).

**Figure 4:**
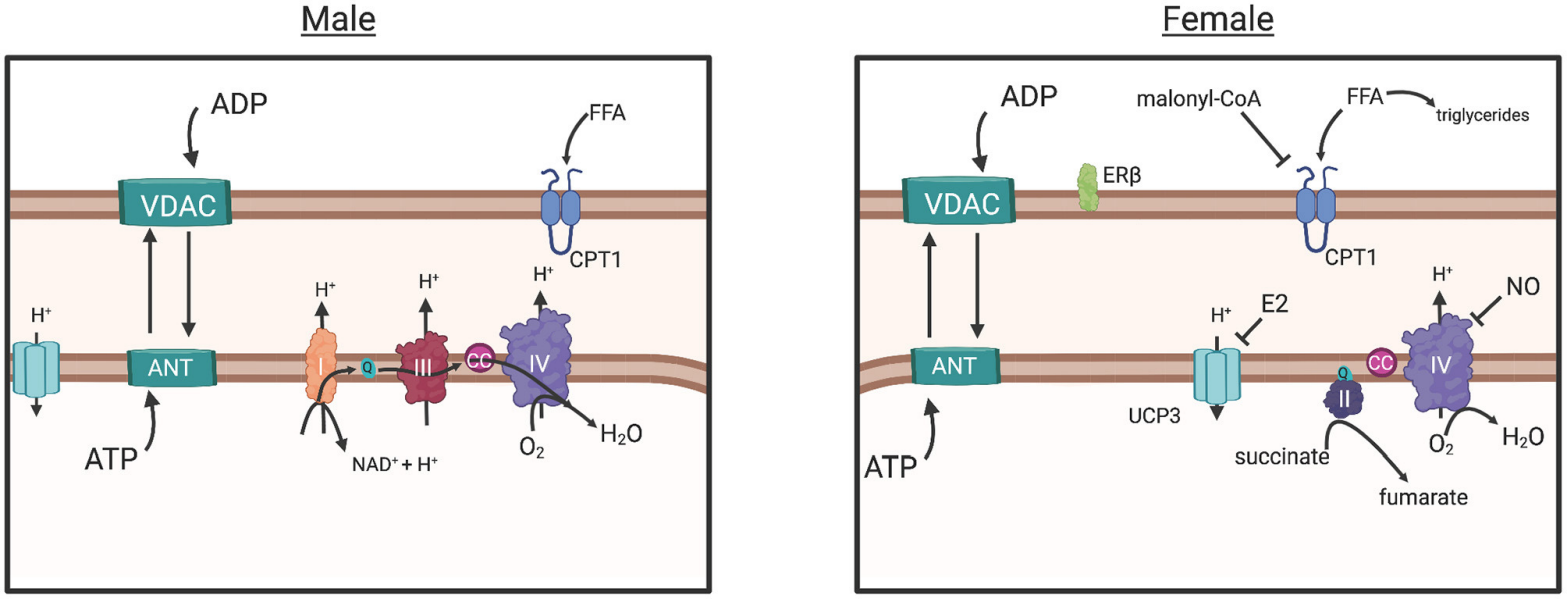
The commonly understood electron transport chain, simplified (male), results in ROS buildup, a consequence of increased O2 diffusion into the mitochondria and subsequent consumption by CIV, greater ADP influx (due to greater ADP sensitivity) and ATP production. The parallel pathway (female) is depicted, however the degree in which it functions over or in conjunction with the common pathway (male) is not fully understood. Briefly: Decreased O2 consumption in peripheral tissues may have induced adaptation of mitochondria in females to require more efficient control of energy production, as evidenced by human and animal studies (in text). Decreased ADP sensitivity (sex differences in VDAC and ANT regulation unknown) in females would prevent large influx of ADP from the cytosol, reducing the production of ROS overall. Estrogen represses Ucp3 expression, preventing energy dissipation, and reduces proton transport into the mitochondria, a possible strategy for energy conservation and a response to decreased ROS production. Increased Malonyl-CoA production, inhibits CPT1 transport of free fatty acids into the mitochondria, resulting in greater triglyceride production in the cytosol and a more controlled energy production within the mitochondria. Greater CII involvement (female), requiring less proton influx may control production of ROS further. Overall, these adaptations suggest that females exhibit an efficient control of ATP production while simultaneously reducing ROS production. Created with BioRender.com. ADP, adenosine diphosphate; ATP, adenosine triphosphate; ANT, adenosine nucleotide translocase; VDAC, voltage-dependent anion channel; CC, cytochrome c; UCP3, uncoupling protein 3; CPT1, carnitine palmitoyltransferase; Complex I, II, II, IV; E2, estradiol; FFA, free fatty acid; ER-beta, estrogen receptor beta; NAD+, nicotinamide adenine dinucleotide; ROS, reactive oxygen species; Q, coenzyme Q; H+, proton.

In another study, intrinsic mitochondrial capacity was higher in endurance-trained females (endurance) when compared to active males with similar VO_2max_, yet similar to males with higher VO_2max_ (Cardinale et al., 2018). However, mitochondrial respiration per unit muscle wet weight was similar between males and females with similar VO_2max_, yet lower in females when compared to males with higher (32%) VO_2max_ (Cardinale et al., 2018). These results suggested that based on a higher intrinsic mitochondrial respiration, females did not need to increase mitochondrial content to the same extent as males to have similar mitochondrial respiration. This study also found potential functional differences in the electron transfer system between males and females, as evidenced by greater intrinsic proton leakage in females and a greater contribution of Complex II in mitochondrial respiratory capacity, a molecular strategy that may assist in reducing ROS-production (Figure 4) (Cardinale et al., 2018). The overall consensus of this study was that the females possessed greater mitochondrial quality than males with similar VO_2max_ and endurance training background, an adaptation that may allow them to counter lower O_2_ transport to peripheral tissues (Cardinale et al., 2018).

The direct role of sex hormones in the function of mitochondria is better understood in animal models than in humans. However, there is strong evidence that supports crosstalk between estrogen signaling and mitochondrial function. For example, a knockout mouse model for an estrogen-induced gene of a nuclear receptor, *NR4A1*, exhibited reduced respiratory capacity of SkM mitochondria through the reduction of ATP production (Chao et al., 2012). Moreover, an exercise response study of castrated male mice participating in an endurance exercise regimen found that androgens, including testosterone, stimulated mitochondrial biogenesis (as measured by oxidative capacity), as mitochondrial biogenesis was reduced compared to non-castrated males (Rossetti and Gordon, 2017). However, how this evidence would translate into exercise differences between males and females needs to be further explored. There is evidence that suggests estradiol localizes to the mitochondrial membrane, resulting in changes to the membrane microviscosity and bioenergetic function (Torres et al., 2017). One mechanism may be through testosterone and 17β-estradiol activation of K+ channels in the inner mitochondrial membrane, resulting in an increase in mitochondrial volume, fatty acid oxidation, oxidative phosphorylation and ATP synthesis (Sakamoto and Kurokawa, 2019). Supporting this, an analysis of differentially regulated genes in muscle suggested that untrained females exhibit higher lipid metabolism relative to untrained males, whereas untrained males exhibit higher expression of factors involved in protein catabolism and peptide metabolic pathways (Chapman et al., 2020). In response to a single acute bout of ET *via* cycling, males receiving 17β-estradiol exhibited higher lipid oxidation and a shift in whole body respiratory exchange ratio (RER) resembling that of females (Hamadeh et al., 2005).

During exercise specifically, females rely more on lipids as fuel (Tarnopolsky et al., 1990, 2007; Venables et al., 2005; Devries et al., 2007; Oosthuyse and Bosch, 2012), perhaps a mechanism of greater type I myofiber percentage resulting in a greater ability for lipid uptake, storage and oxidation than males (Malenfant et al., 2001). This is also supported by higher expression of β-oxidation enzymes for long-chain fatty acid (FA) oxidation in the SkM of women (Maher et al., 2010). However, identifying the precise location of FA origin for this fuel has been difficult. For example, while several studies were unable to identify sex differences in plasma FA utilization during exercise particularly when VO_2max_ is normalized with lean body mass (Romijn et al., 2000; Beaudry and Devries, 2019), one study identified the increased use of plasma FA (47%) in females after 30 min of cycling (Roepstorff et al., 2002). Additionally, the question of whether lipid reliance seen in females is based on intramyocellular lipids (IMCL) content remains controversial within the literature (Beaudry and Devries, 2019). IMCL plays a crucial role in SkM metabolism by contributing an intracellular source of energy to the working muscle independent of glycogen use (Beaudry and Devries, 2019). During IMCL utilization, FAs are transported into the mitochondria for oxidation to generate energy for the working muscle or used to produce triglycerides to regulate FA delivery to the mitochondria (Figure 4) (MacPherson and Peters, 2015). Females have more IMCL storage than males, influenced by estrogen, particularly in Type I myofibers (Devries et al., 2007). With increased Type I fibers and increased IMCL content in females, metabolic pathways associated with FA uptake, storage, and utilization are typically upregulated in females (Beaudry and Devries, 2019). For example, CD36, a transmembrane protein involved in lipid metabolism, is expressed at higher levels in females, regardless of training status (Kiens et al., 2004; Miotto et al., 2018). However, while evidence suggests that females rely on fatty acids as a metabolic substrate, they are also more sensitive to inhibition of fatty acid synthesis through malonyl-coA within SkM (Miotto et al., 2018). Malonyl-CoA inhibits CPT1, the enzyme that controls long-chain fatty acid transport into the mitochondria for oxidation (Figure 4) (Ruderman et al., 2003). This is thought to be a mechanism of why females have higher rates of free fatty acid incorporation into triglycerides and higher body fat percentage (Ferreira, 2018).

It is noteworthy to mention that in response to long-term endurance exercise, several sex differences may be somewhat diminished as long-term endurance training enhances lipid metabolism in male athletes and protein catabolism in females (Chapman et al., 2020), although some evidence exists that long-term trained females still prioritize fat oxidation vs. males (Montero et al., 2018). Overall, the influence of these metabolic mechanisms on exercise response, particularly between the sexes, remains both controversial and limited in evidence and is an area that is in need of further exploration.

#### Sex Differences in Muscle Damage Inflammation and Repair

SkM regeneration is a key process in exercise adaption and muscle growth. Driving this process is exercise-induced inflammation which presents in phases to clear the tissue of exercise-induced damage, stimulate satellite cell growth and differentiation, as well as myocyte protein synthesis. Ultimately exercise-induced inflammation is key in repairing and building SkM (Chazaud, 2016; Peake et al., 2017). While this process exists in both males and females, there may be a few differences during this process that dictate the extent of the inflammatory response and muscle adaptation, due to the relationship of female sex hormones and immune function (Giraldo et al., 2008). For example, estrogen has been correlated with higher humoral immune function while sex hormones progesterone and testosterone have been associated with immune-suppressing effects (Giraldo et al., 2008). Whether this is similar in exercise-induced inflammation and muscle regeneration remains controversial. In addition, it is important to mention that some of the observed sex differences in muscle regeneration can also be independent of hormone influences, but dependent on the original sex of the cells themselves and the interplay of the sex chromosome complement with the tissue environment (Rich-Edwards et al., 2018). For example, muscle stem cell transplantation in male and female mice exhibited enhanced regeneration if the stem cells were from female mice (Deasy et al., 2007). Therefore, while much of the conversation revolves around hormone-influenced sex differences, the innate sex differences of muscle cells may also play a crucial role in muscle repair and regeneration.

Within the muscle, during the exercise-induced inflammatory response, the MPC pool is expanded to meet the needs of muscle repair and regeneration (Adams, 2006). A key sex difference seen in this ability is expansion capacity, which is greater in males than in females (Riddle et al., 2018). A factor in this may be the oxygen consumption rate of MPCs, an indicator of oxidative phosphorylation which is seen to be lower in females than in males (Riddle et al., 2018). In addition, satellite cell differentiation capacity in male mice has shown greater myogenic differentiation in the presence of low oxygen and induced oxidative stress (H_2_O_2_) (Deasy et al., 2007). In contrast, during this type of stress, cells from female mice remain undifferentiated until acute inflammation subsides, at which point the female cells begin expansion (Deasy et al., 2007).

There is evidence that suggests that premenopausal females have an overall attenuated inflammatory response when compared to males during exercise, possibly dependent on menstrual cycle phase or specific hormones (i.e., estrogen) (Stupka et al., 2000). For example, transcriptomic evidence has suggested that in response to long-term endurance training males exhibit higher expression of inflammatory pathways while females have attenuated expression (Chapman et al., 2020). One potential mechanism identified in a rat model, is the inhibition of the inflammatory response *via* estrogen through reductions in neutrophil infiltration post-exercise (Tiidus et al., 2001). In human studies, decreased focal damage, bcl2- and LCA-positive inflammatory cell infiltration post-exercise has also been observed in muscle from females when compared to males (Stupka et al., 2000; Tiidus, 2003). In addition, a clinical study utilizing hormonal therapy in post-menopausal females also observed attenuated SkM damage, further demonstrating the immune modulating and protective role of estrogen (Dieli-Conwright et al., 2009). The process through which estrogen may modulate this reduced inflammatory response is through its antioxidant and membrane stabilizing characteristics, resulting in protection against peroxidation (Kendall and Eston, 2002).

In circulation, granulocytes and some cytokines (i.e., IL-1, IL-6) have been shown to increase in males, and not females, post-eccentric muscle damaging protocol (Stupka et al., 2000). Furthermore, females well-trained in resistance exercise (5.5 +/- 4.9yr) exhibited little to no significant change in blood markers of inflammation (IL-6, TNFα, and CRP) induced by muscle damage following eccentric squats regardless of their menstrual cycle phase (Romero-Parra et al., 2020). In contrast, professional female soccer players subjected to a maximal endurance protocol, exhibited higher circulating levels of IL-6 immediately post-exercise but displayed an inverse relationship based on training experience (Janikowska et al., 2020). However, overall, there were no significant changes in circulating IL-1β and TNF-α in this study, leading the authors to suggest that the athletes may not have experienced a systemic inflammatory response or muscle damage, although analysis of muscle itself was not conducted (Janikowska et al., 2020).

Circulating creatine kinase (CK), often used a measure of tissue damage, has been shown to be differentially expressed in males and females post-resistance exercise (90% 1-RM) (Wolf et al., 2012). Specifically, elevated CK levels have been shown in males up to 24 h post-resistance exercise, while females continued to exhibit levels similar to pre-exercise (Wolf et al., 2012). Together the muscle- and circulation-derived data discussed here (i.e. low circulatory inflammatory response and low inflammatory cell infiltration in tissue) suggests that females may exhibit an attenuated inflammatory response to exercise (although, not conclusive), or at the least during the time points sampled in these studies. Understanding how this may impact muscle repair and regeneration between the sexes may be of great interest and is an area that needs more focus, particularly if the data could guide development of exercise regimens to enhance muscle hypertrophy and overall performance.

It must be stated that hormonal (growth, stress, and sex hormone) reference ranges vary between the female general population and the athletic population, contributing to confounding understanding of hormonal influence in exercise in either population, particularly in stress response, inflammation and muscle adaptation (Roli et al., 2018). As mentioned earlier, hormone-associated data derived from animal models may be speculatory at best due to the differences in reproductive cycles and hormone response between species (Hansen and Kjaer, 2014). In addition, many of the studies here discuss reduced inflammatory responses in females that are within the follicular phase. This results in a skewed perception of exercise-induced inflammatory response between males and females, as some evidence suggests that females are more susceptible to exercise-induced inflammation during the luteal phase (at least with endurance exercise), suggesting that further research is warranted to understand inflammatory responsive shifts throughout the menstrual cycle (Northoff et al., 2008).

### Additional Sex Differences to Consider in Exercise Response

#### Cardiopulmonary

Response to exercise is greatly influenced by the pulmonary system's morphology and function. Historically, much of the early studies have predominately utilized males to identify the pulmonary system's contribution to the integrative exercise response. However, there are notable differences between males and females in pulmonary structure that translates into differences of functional capacity. Briefly, the pulmonary system consists of lungs, airways, rib cage and respiratory muscles. When compared to males, females have smaller lungs overall (matched to height) (Crapo et al., 1982; Schwartz et al., 1988) with a prismatic lung and rib cage shape (Torres-Tamayo et al., 2018), smaller airways (when matched for lung size) (Sheel et al., 2009), large conducting airways (Dominelli et al., 2018), and lower number of alveoli (relative to lung size) (Sheel et al., 2009). While breathing mechanics are not different between the sexes at rest, the response to exercise diverges (Molgat-Seon et al., 2018b). Airway morphology determines the mechanical work needed to achieve ventilation through viscoelastic and resistive forces and as a result of smaller airways, females have a higher mechanical workload threshold (due to differences in absolute body and lung size) to achieve a given minute ventilation (Wanke et al., 1991; Molgat-Seon et al., 2018a) regardless of training status and age (Dominelli et al., 2019). This then translates to an increased oxygen uptake (VO_2_) by respiratory muscles, where it has been observed that at maximal exercise (cycle ergometer) females dedicate on average 5% more of total body VO_2_ to the respiratory muscles (Dominelli et al., 2015). Interestingly, even with the increased pulmonary workload seen in females, diaphragm fatigue has been observed to be significantly greater in males (Guenette et al., 2010). This contradictory result may be due to differential recruitment of inspiratory muscles at maximal exercise: males tend to rely on their diaphragm to a greater degree while females may recruit extra-diaphragmatic inspiratory muscles (e.g., scalene and sternocleidomastoid) (Guenette et al., 2010; Mitchell et al., 2017). However, more research is needed in this area to fully elucidate the mechanisms responsible and whether differential training strategies (i.e., resistance vs. endurance) might promote enhanced exercise capacity between the sexes.

Cardiovascular adaptations in females have been shown to present differently in response to endurance training. Specifically, males (vs females) seem to have greater adaptations in left ventricular mass (relative to body mass), stroke volume, and VO_2max_ (relative to baseline body surface area) after 1 year of ET (Howden et al., 2015). Medically, it is understood that females are at lower risk for cardiovascular diseases up until menopause, which has been attributed to ovarian hormones, particularly estrogen (Colditz et al., 1987). Specifically, cardiovascular burden may be lower in females due to their overall lower blood pressure (BP) and ability to control the extent of vasoconstriction *via* β-adrenergic receptors compared to males (Collier et al., 2011; Hart and Charkoudian, 2014). However, the literature remains controversial on exercise BP response, with evidence showing no difference during exercise (Maruf et al., 2012), and other showing greater BP in males (Dimpka et al., 2008). Post-exercise, females tend to exhibit greater decreases in BP (Carter et al., 2001a; Senitko et al., 2002; Wee et al., 2018; Bassareo and Crisafulli, 2020). For example, in a study observing post-high-intensity exercise BP, recovery tended to be quicker in females suggesting greater vasodilatory effects and as a result quicker vascular recovery (Marshall et al., 2021). Nevertheless, the evidence supporting this may still be debatable due to differences in measurements of BP control within these studies, especially since BP has been shown to be regulated through both central and peripheral mechanisms (Senitko et al., 2002; Hart and Charkoudian, 2014), of which sexual dimorphism has been observed. Specifically, BP control post-exercise in men seems to be centrally regulated *via* stroke volume (Queiroz et al., 2013; Wee et al., 2018) while females tend to regulate post-exercise BP through peripheral vasodilation (Senitko et al., 2002; Parker et al., 2007; Queiroz et al., 2013; Hart and Charkoudian, 2014), although such regulation may be dependent on mode and intensity of exercise (Rossow et al., 2010).

Another notable difference in the cardio-pulmonary system is hemoglobin content. Males, on average, have 12% more circulating hemoglobin (g/dL) (Murphy, 2013), thought to result in greater oxygen transport (Ekblom et al., 1972), uptake, (Sparling, 1980) and thus greater exercise capacity. However, females have greater peripheral vasodilation and number of red blood cells per unit of blood within the microvasculature, and thus better muscle perfusion (Parker et al., 2007; Murphy, 2013; Kellawan et al., 2015), a mechanism that in part may contribute to muscle hypertrophy in females (Dankel et al., 2016). This greater vasodilation in response to exercise can also be observed even when females are tested during menstruation (low estrogen), indicating a sex difference dependent on a non-hormonal mechanism (Kellawan et al., 2015).

#### Hepatic

The liver is a major mediator of muscle response to exercise, coupled with adipose tissue. Sexual dimorphism in the liver is thought to be driven by estrogen, as hepatic estrogen receptor expression is second only to gonadal expression (Roy and Chatterjee, 1983). In addition females exhibit 38% greater liver volume per unit of lean body mass (kg) when compared to males (Kwo et al., 1998). The biological importance of estrogen in the liver is evidenced during the initial stages of pregnancy when the female liver dramatically shifts lipid production for support and storage (Woollett, 2011; Baardman et al., 2013). Additionally, females are more efficient at clearing fat from the liver through oxidation and triglyceride secretion in the post-prandial state, which is associated with an increased liver production rate of the ketone, 3-hydroxybutyrate (Pramfalk et al., 2015).

In response to exercise, the liver is an important mediator of lipid and glucose metabolism, particularly as a source of energy for muscle contraction (Gonzalez et al., 2016). Sex specific differences in hepatic lipid contribution to muscle during exercise may not be apparent or fully elucidated, indicating that this may be an area in needing more research in light of the considerable differences of liver function between males and females. However, it is generally understood that females have a reduced reliance on glycogen and preferentially utilize lipids as a source of energy metabolism at any given exercise intensity (Tarnopolsky et al., 1990, 2007; Venables et al., 2005; Devries et al., 2007; Oosthuyse and Bosch, 2012). Yet, this energy preference does not necessarily translate to greater lipolysis in females compared to men and may be exercise mode specific. A recent study by Forsyth and Burt (2019). concluded that fat oxidation rates post-sprint interval training was higher in males than in females (when matched for physical activity), a result that supports evidence suggesting females have greater metabolic control resulting in quicker return to resting metabolic values, resulting in overall reduced levels of fat oxidation (Esbjörnsson-Liljedahl et al., 2002; Henderson et al., 2007; Henderson, 2014), Still, when controlled for fat-free mass (FFM), these sex differences are reduced suggesting fat metabolism can be reliant, in-part, on FFM (Forsyth and Burt, 2019), Overall, given the liver's role in energy balance, more research regarding its role in exercise response in both males and females needs to be further explored.

## Considerations for Practical Application

### Tailoring to Sex in Athletes

Historically, males have outperformed females in most sports, a result that is often credited to increased body size, speed, and strength (Handelsman et al., 2018). As more females gained access and began participating in sport post Title IX, the gap in performance has continually decreased (Thibault et al., 2010). Regardless, sex differences in sport remain which may be attributed to differences in physiology when all other components (i.e., access to and quality of training) are the same. A recent review by Landen et al. covers in-depth the physiology and molecular sex differences in a performance light (Landen et al., 2021). In regard to optimizing training based on sex differences, most of the current literature centers on the female athlete and energy balance, particularly nutritional requirements to maintain energy balance for optimal performance and recovery (recently reviewed by Wohlgemuth et al., 2021). Instead, herein, we focus on sex-specific training aspects that may enhance or inhibit optimal recovery, muscle adaptation and performance in athletes.

#### Sex Differences in Military Training Response

Basic and career-long military training, unlike athletics, focuses on task-oriented performance that includes a multitude of environmental and physical stressors (Gold and Friedman, 2000) studies have reported sex differences in the response to military training with express concerns of how training can be optimized to prevent injury, especially in light of reports that by completion of basic training, 50% of females experience one or more injuries, including bone stress fractures, and are more likely to sustain injury when compared to males (Friedl et al., 2008; Gill et al., 2021). This may be due to a mixture of anatomical, biomechanical, and physiological differences between males and females. For example, a U.S. military study found that female soldiers had greater balance, flexibility, range of motion in upper and lower extremities, knee flexion and greater hamstring flexibility, whereas, male soldiers had greater strength, anaerobic, and aerobic capacity, but lesser range of motion and flexibility (Allison et al., 2015). In this study, sex differences in strength (i.e., shoulder, knee, ankle, torso strength) were mitigated when the top-performing percentile of females were compared to the lower percentile of men, as indicated by these females exhibiting similar or better strength characteristics (normalized to body weight) (Allison et al., 2015). Furthermore, the biomechanical adaptation to military training loads between the sex presents differently: females have been shown to undergo kinetic changes at the hip and kinematic changes to the knee, possibly an adaption to heavy loads that contributes to increased injury susceptibility in the knee (Loverro et al., 2019), An extensive review by Nindl et al. reported that a regimented physical training program, including resistance and endurance training, was superior for female military personnel in developing higher levels of strength and endurance than basic training (Nindl et al., 2016), and is likely applicable to both sexes. Continuing to fortify our understanding of how to optimally train soldiers of both sexes based on musculoskeletal anatomy along with a range of other factors contributing to physical resilience is of great interest.

#### Considering the Impact of Sex Differences on Athlete Health and Performance

Training and recovery are essential for athletic performance and, when not optimal, can cause reductions in performance and health (i.e., overreaching, overtraining syndrome). While there has not been extensive research in sex differences regarding optimal training approaches, or even if it should be approached differently, some physiological aspects may suggest that males and females would benefit from more personalized approaches. For example, males and females exhibit differences in muscle, tendon, and ligament injuries (Chidi-Ogbolu and Baar, 2019). In addition, hormonal fluctuations throughout the menstrual cycle are thought to influence performance in athletes (Nattiv et al., 2007).

Menstrual abnormalities in female athletes are a major contributor to decreased health and performance and range from infrequent menstrual periods (oligomenorrhea), the absence of ovulation (anovulation), or the absence of a menstrual period entirely (amenorrhea) (Souza et al., 2004). These conditions are typically multifaceted and are not caused by excessive exercise alone, but include disordered eating driven by societal and internal pressures to maintain a low body weight or body fat percentage for competition (Souza et al., 2004). This chronic energy deficit leads to decreasing concentrations of estrogen that lead to menstrual abnormalities, low bone mineral density (BMD), osteoporosis, and premature cardiovascular disease, all which can severely impact sport performance (Nattiv et al., 2007; Areta et al., 2021). For example, a study comparing eumenorrheic and amenorrheic athletes found lower neuromuscular performance, fat free mass, estrogen, triiodothyronine (thyroid hormone, T_3_) in amenorrheic athletes (Tornberg et al., 2017). It has become increasingly clear that a functional menstrual cycle needs to be considered a major health marker for female athletes.

Excessive or chronic exercise can lead to decreased performance in both males and females. Most of the research in overreaching and overtraining syndrome (OTS) have centered around endurance exercise. Generally, overreaching is a short-term phenomenon, while overtraining is the addition of an additional stressor (i.e., hormonal dysfunction, chronic inflammation, etc). However, the specific underlying characteristics are still not completely understood (Kreher and Schwartz, 2012). For example, a study in male and female triathletes and cyclists following either 100% or 150% of their normal training routine found, that over the course of 3 weeks, those training at 150% exhibited in reduced peak power from pre- to post- training (Coates et al., 2018). Notably, the overtrained group exhibited increased arterial stiffness, decreased maximal heart rate, cardiac output and stroke volume when compared to the control group. Sex differences were not analyzed in this study as it was underpowered. In resistance training, the occurrence of either syndrome is not as well understood, and it has recently been reported that there is little evidence of true OTS occurrence in strength sports (Bell et al., 2020; Grandou et al., 2020). However, non-functional overreaching in resistance training has been observed in chronic high-volume or high-intensity conditions (Bell et al., 2020). Overall, the specific presentation of OTS differences between males and females has not been specifically researched as there are several training models that either geared specifically toward females (TRIAD) (Nattiv et al., 2007), both (RED-S), (Mountjoy et al., 2014) or males (EROS) (Cadegiani et al., 2020). It would be pertinent to analyze whether there are exercise- and/or sex-specific drivers in overtraining presentation and whether the approaches to mitigate the occurrence should be different between the sexes.

Males and females exhibit differences in muscle, tendon, and ligament injuries (Chidi-Ogbolu and Baar, 2019). Specifically, males tend to have more muscle injuries while females to have more ligament injuries (reviewed in Chidi-Ogbolu and Baar, 2019), thought to be connected to estrogen's effect on ligament softening, particularly noticeable in greater anterior cruciate ligament (ACL) injuries (Lin et al., 2018; Chidi-Ogbolu and Baar, 2019). Another consideration in differences in injury rates can be related to training approaches. In a recent study in rugby players, there was a greater overall injury rate among males; however, 83% of injuries in females occurred in the later half of game play (concussion and ACL injury). The authors postulate a mixture of inadequate training preparation coupled with in-game fatigue resulted in this higher incidence and suggest that more emphasis on strength and conditioning could improve these rates (Yeomans et al., 2021). Indeed, ACL injury prevention programs for female athletes can improve injury rates, although the type of sport may require different approaches (Michaelidis and Koumantakis, 2013; Nessler et al., 2017). Additionally, consideration must be given to the impact of low energy availability and other factors on injury risk and perhaps the injury rates currently seen are underpinned by energy deficiency (Mountjoy et al., 2014) and that with adequate care, nutritional support and training, these injury rates may improve for both males and females.

Overall, given the importance of the menstrual cycle and estrogen in exercise response and performance in females, it seems imperative to stress their preservation in female athletes. Further research is needed to determine whether training approach (in the absence of energy deficiency) may be contributing to menstrual cycle abnormalities and injury rates. Specifically, are females following training regimens designed by males for males at a greater risk of reduced performance or less optimal adaptions to the training due to their physiology? Would designing training regimens that synchronize with changes in the menstrual cycle will reduce the risk of injury, for example, with a focus on technique around ovulation (high estrogen) and maximizing performance adaptations during the follicular phase? These questions would require far more targeted research in athlete populations to become better understood.

### Tailoring to Sex in the General Population, Untrained and Recreationally Active

When discussing health and wellness, the target population is the general population or individuals seeking recommendations for maintaining a healthy lifestyle. The ACSM guidelines for endurance and resistance training were established in 1998, and current recommendations are set at 3–5 days per week of endurance training at moderate- to vigorous-intensity (46–63% of maximal effort) for a minimum of 150 total min and 2–3 resistance training session per major muscle group per week (Garber et al., 2008). Overall, the recommendations are intended for males and females, as well as some chronic disease populations, children, and pregnant females with minor adjustments. Here we discuss sex differences in exercise response in the untrained and recreationally active (non-competitive) populations for considerations in targeting elements of exercise prescription to sex.

#### Untrained Individuals

Many exercise response studies utilize untrained individuals and document changes associated with participation in regimented exercise. In addition, in order to consider sex differences in approaches to regimented exercise, the training modality itself must be considered as this is associated with specific adaptations and effort (American College of Sports Medicine, 2009). For example, a study of untrained females and males that underwent identical endurance training for a year reported blunted increase in left ventricle (LV) hypertrophy and VO_2max_ in females (relative to fat free mass). This was mainly due to females reaching their maximal LV mass and VO_2max_ after 3 months of training (Howden et al., 2015), suggesting that these females adapted to the training quicker than their male counterparts, then reached a plateau. Continually modifying the exercise dose may have encouraged continual adaptation in the female participants. On the other hand, the males exhibited larger diastolic reserve after 1 year of training, allowing higher stroke volume and cardiac output when compared to the female participants, while ventricular compliance and distensibility was similarly improved between the sexes (Howden et al., 2015; Diaz-Canestro and Montero, 2020).

In regards to RT, a study focusing on multi-joint vs. single joint RT regimens in untrained females, found no differences in strength and muscle gains between the groups (Barbalho et al., 2020). Similar results have been reported for untrained males (Gentil et al., 2013). In contrast, a recent meta-analysis reported that, while gains in strength and hypertrophy were similar between the sexes, greater strength changes were observed for the upper body in untrained females (Roberts et al., 2020). Furthermore, a recent meta-analysis of RT effects in untrained females reported that the variables most effective at increasing strength were training frequency and volume. Overall, it was suggested that regimented exercise should be performed at 3–4 sets/exercise, 2–4 days/wk for optimal strength adaptations (Hagstrom et al., 2020). It is worth noting that the authors were unable to find a strong relationship between exercise variables (i.e., load, volume, sets) and magnitude of hypertrophy and discovered a lack of significance in hypertrophy gains between high and low intensity training, although more research is needed as the majority of studies are limited to durations <12 week (Hagstrom et al., 2020). However, these studies suggest that untrained individuals can experience significant muscle adaptations in RT without large time commitments, a key variable in establishing exercise adherence in the untrained population.

It is worth noting, cycling hormones throughout the menstrual cycle can have an impact in exercise response, this may be a clear sex difference to consider in untrained females. For example, in a report by Sung et al. (2014), untrained females undergoing a three-month RT regimen exhibited significantly increased maximum isometric muscle strength (one-leg knee extension) during the follicular phase when compared to the luteal phase. This was further supported by significant increases in Type II fiber diameter and nuclei-to-fiber ratio only after training in the follicular phase, with no significant improvements after training in the luteal phase (Sung et al., 2014). While a single study, the results suggest that designing a training regimen alongside the menstrual cycle would be beneficial for optimum exercise response in untrained females, although longer-term research looking at these specific parameters is warranted to rule out short-term exercise adaptations.

#### Recreationally Active

A recent study examining resistance-trained males and females with a minimum of 3 years of experience (3 or more d/wk), demonstrated that trained females fatigued slower than males, similar to previous research in untrained individuals (Metcalf et al., 2019). However, a notable sex difference observed in untrained individuals (Martin and Rattey, 2007) that disappeared in these trained individuals was that central motor output was not affected even while muscle contractility declined throughout the sessions (Metcalf et al., 2019). Similarly, a study examining mechanisms of fatigue in 17 females and 14 males in response to fatiguing sustained isometric knee extension demonstrated that fatigability in males during the exercise was associated supraspinal fatigue and not twitch amplitude of muscle fibers. On the other hand, dynamic fatiguing knee extensions resulted in lowered twitched amplitude in males vs. females without supraspinal fatigue during the recovery period (Senefeld et al., 2018). In addition, recovery from strength training is similar to endurance training, wherein females also demonstrate quicker recovery from maximal exercise bouts. It is important to mention that there haven't been any apparent sex differences when examining strength losses post-exercise, but rather a difference in recovery capability, suggesting that females are able to return to a functional level quicker than males, (Häkkinen, 1993; Sayers and Clarkson, 2001), although there is evidence of reduced recovery when estrogen levels are higher (Markofski and Braun, 2014), and some measurements (knee extensor strength and countermovement jump height) of neuromuscular recovery may be better in males (Davies et al., 2018). Altogether, differences in mechanisms of fatigue and recovery not only seem to depend on sex, but also the type of fatiguing task. More research is needed to determine that taking these differences into account for the development of exercise regimens would result in optimized performance.

### Sex Differences in Successful Aging and Exercise Approach

Prescriptive exercise training and general physical activity promotion are both receiving greater emphasis in disease prevention and treatment, and in the promotion of healthy longevity throughout the lifespan. A perhaps overlooked consideration is the differential physiological trajectory of aging in females vs. males. Starting at puberty, hormones are the primary contributor to the divergence in exercise performance and adaptations seen in children. Over the course of a lifetime, males typically experience a steady hormonal state until declines begin in the mid to late thirties, and even so, often do not experience drastic declines. In contrast, eumenorrheic females experience cyclical changes in hormones, potentially interrupted only by major events such as pregnancy and the postpartum period, until perimenopause (period of time where hormones decline up until menopause occurs) and eventual menopause (marked by 12 months of no menstruation), in which a new hormonal norm is established. Current exercise recommendations are set as rough guidelines to maintain health and are not tailored to the needs of an individual at a given life stage. However, just as transient changes in female hormones influence the exercise response, changes across the lifespan should be considered as critical variables in designing an appropriate exercise regimen.

Ideally, to promote an active lifestyle aimed at ensuring health and longevity, participation in regimented exercise would begin in children. After puberty, the major hormonal alteration experienced in females (sans pregnancy) during the advancement of age is perimenopause, which is paralleled by declines in testosterone in some males (Vingren et al., 2010). Accompanying these hormonal declines is the loss of muscle mass and strength, with sex differences demonstrated in the changes in muscle composition, strength and exercise response. For example, estrogen normally decreases bone reabsorption and maintains mechanosensitivity, which during aging starts to decline along with the estrogen receptor alpha (ERα), causing a decreasing muscle and bone responsiveness to exercise by reducing the ability of exercise to stimulate an osteogenic response (Lee and Lanyon, 2004; Lang, 2011). In addition, with age, female Type II muscle fibers tend to atrophy and reinnervate leading to Type I fiber grouping while male muscle loses overall myofiber number, including Type II fibers, the remaining of which hypertrophy to compensate for the fiber loss (Roberts et al., 2018). This is further supported by a mitochondrial DNA deletion mutation rate, which is higher in aging males (sedentary), and results in the activation of apoptosis leading to muscle fiber loss (Herbst et al., 2021). Thus, aging males experience a greater loss in strength and muscle mass than females (both absolute and relative to body mass), which indicates females maintain muscle quality better (Janssen et al., 2000; Goodpaster et al., 2006; Roberts et al., 2018). Nonetheless, males preserve bone better with age, although, the loss of androgen signaling in older men may also decrease the efficacy of resistance training on bone (Lang, 2011).

Masters athletes or individuals that continue participation in systematic training and competition (Tanaka and Seals, 2008), are of great interest to the medical community as these may be the closest example of “successful aging” that occurs in the population. Furthermore, how continued exercise affects either sex throughout age may highlight important strategies maintaining health and longevity for both sexes. While athletes tend to exhibit minimized sex differences than what is seen in the general population, performance during aging may decline in a sex-specific manner. On a molecular level, a recent study of master's endurance athletes aged 44–83 years old, reported no significant sex differences in rate of decline of neuromuscular function and motor unit (MU) remodeling (based on needle electrode analysis of mid-level contractions); however, MU firing rates decreased more substantially in females more than males, perhaps reflecting a greater tendency to shift to slower twitch fibers (Piasecki et al., 2021), evidence that our lab has also reported on (based on fiber type grouping) (Roberts et al., 2018). Overall, master's athletes tend to better conserve several markers for high physical function (i.e., muscle mass and capillarization, oxygen uptake, mitochondrial biogenesis, etc.) (Geard et al., 2017), further providing a strong argument for continued exercise for SkM health throughout the aging continuum for both sexes.

After menopause alters the hormonal landscape in females, the result is reduced protection against chronic diseases and simultaneously accelerated muscle protein synthesis and breakdown (Smith et al., 2014). It is thought that declines in estrogen cause an imbalance in the anabolic/catabolic pathways, allowing for catabolic pathways to tip the scales into loss of muscle mass (Chidi-Ogbolu and Baar, 2019). This hypothesis is further supported by research with estrogen replacement therapy, demonstrating exogenous estrogen is able to normalize anabolic response to exercise in post-menopausal females (Pöllänen et al., 2010; Hansen et al., 2012), perhaps even contribute to better muscle hypertrophy (Dam et al., 2021).

The literature specifically pertaining to sex differences in exercise response within older cohorts is contradictory at best, with several studies noting significant differences in muscle mass and/or strength (Bamman et al., 2003; Boit et al., 2016; Stec et al., 2017) and others finding none (Leenders et al., 2013; Miller et al., 2021). These results may be due to a combination of differences in cohorts, exercise regimens, exercise choice, length of interventions, as well as, physiological status of sex hormones. However, the general consensus is that on a full body level (i.e., strength, hypertrophy), older males and females enjoy similar long-term muscular adaptations, supporting the rationale that RT remains the best approach to preventing age-related declines in muscle mass and strength during aging (Miller et al., 2021). Even so, the possibility that older females may respond more efficiently to lower RT exercise frequency has been suggested, as evidenced by significant fiber adaptations with progressive training over the course of 6 months when compared to male participants (Häkkinen et al., 2001). Therefore, designing RT approaches that allow for the maximization of the muscle females tend to maintain as they age (hypertrophy and strength) may be a separate approach than for males where exercise interventions could be geared toward prevention of fiber loss through more frequent RT bouts.

## Discussion

The literature demonstrates differences in female and male anatomy, physiology, and SkM to the extent that exercise responses may diverge based on these factors. In addition, a repetitive phenomenon was observed in the literature that suggests true sex differences may be uncovered in individuals who are normally and highly active (refer to Figure 3). However, the literature on sex differences in strength and hypertrophy gains remains incomplete. This is likely due to a mixture of lower numbers of female recruitment resulting in the inability to plan for sex differences analyses, underpowered *post-hoc* analyses for sex differences, and the lack of adequate representation of females across all phases of the menstrual cycle and all levels of physical activity. Further research is needed that directly compares the typical exercise approaches to approaches designed around the menstrual cycle. Optimizing training regimens for females based on the menstrual cycle may help decrease injury rates and metabolic burden associated with approaches traditionally designed and utilized by males.

Many of the studies here were chosen to highlight differences that have been discovered between males and females in respect to exercise response. While some of these studies highlight discoveries in their infancy, we found it pertinent to discuss them as areas where more research and follow-up is needed. Much of the established literature demonstrates that males and females have similar outcomes on a full-body or macro scale (i.e., strength, hypertrophy, power, VO_2_) (Walts et al., 2008), particularly when normalized to lean mass or SkM mass. This review highlights, that while most phenotypic adaptations to exercise are conserved between sexes, there are many mechanistic differences that have been described, but incompletely explored. The means by which some of these outcomes are achieved on a molecular level may be different (also known as latent sex differences), thus there needs to be concerted efforts to determine the sex specific underpinnings to exercise induced adaptations (Beltz et al., 2019). Latent sex differences are vital in considering drug response or even using the molecular data we collect to identify viable drug targets, for example, sex differences in receptors that bind to opioids for efficient pain treatment, hepatic enzymes resulting in differences in drug metabolism, and many more thoroughly reviewed recently (Waxman and Holloway, 2009; Farkouh et al., 2020; Lopes-Ramos et al., 2020; Bhargava et al., 2021). Although, we may not expect detrimental outcomes from exercise (though, it is possible), as has been highlighted in drug response, it is still crucial to understand the differences in exercise response to optimize approaches that are applicable for both or either sex. Thus, highlighting physiological and molecular sex differences could mean a difference of efficacy in exercise approaches.

Of importance, the language and tone of sex-cognizant research must be conveyed appropriately. Care must be taken not to depict either sex as “at fault” for not measuring up to the other. Certainly, a reference is needed, but the implication that a different response is a “wrong” response is not only misguided but likely harmful to the perception of sex differences in scientific research. Further it may overshadow crucial information that is directly applicable to females and training optimization. Sex-specific adaptations should be considered on their own sex-specific scale, enabling a clearer distinction of a healthy reference that is not influenced by sex-biased data. Such steps will help prevent misinterpretation of data due to improper scaling or reporting of absolute differences between the sexes.

Overall, there are clear sex differences that impact exercise response, particularly at the molecular level. Specifically, hormone and metabolic states play major roles in the exercise response and suggest that males and females may benefit from exercise approaches optimized to sex. As of now, it would be difficult to claim that exercise strategies that have been deemed effective in males are equally effective in females. As it stands, no area of research in exercise biology and medicine has adequately studied females, particularly in light of the plethora of research on males. Therefore, it is of utmost importance to continue to focus efforts toward increasing the recruitment, retention, and inclusion of females in exercise research, as this remains a major limitation in the field. Furthermore, studies underpowered for sex differences should include sex-specific data within publications or as supplementary material instead of attempting to analyze and overinterpret potential differences. These modifications can substantially advance the field's ability to understand and appreciate potential sex differences toward optimizing exercise prescriptions for health and performance in both sexes.

## Author Contributions

SO'B is the main author responsible for this paper, contributing to the conceptualization, and majority writer. KC was responsible for athlete focused contributions in Section Considerations for Practical Application of the manuscript. DD and KL provided expertise during review and revisions and their input help structure the manuscript as seen now. MB is the corresponding author and mentor to SO'B. All authors contributed to the article and approved the submitted version.

## Conflict of Interest

The authors declare that the research was conducted in the absence of any commercial or financial relationships that could be construed as a potential conflict of interest.

## Publisher's Note

All claims expressed in this article are solely those of the authors and do not necessarily represent those of their affiliated organizations, or those of the publisher, the editors and the reviewers. Any product that may be evaluated in this article, or claim that may be made by its manufacturer, is not guaranteed or endorsed by the publisher.
